# Imaging blood–brain barrier disruption in neuroinflammation and Alzheimer’s disease

**DOI:** 10.3389/fnagi.2023.1144036

**Published:** 2023-03-17

**Authors:** Rae-Ling Lee, Kristen E. Funk

**Affiliations:** Department of Biological Sciences, University of North Carolina at Charlotte, Charlotte, NC, United States

**Keywords:** aging, inflammation, magnetic resonance imaging, neurodegenerative diseases, neurovascular

## Abstract

The blood–brain barrier (BBB) is the neurovascular structure that regulates the passage of cells and molecules to and from the central nervous system (CNS). Alzheimer’s disease (AD) is a neurodegenerative disorder that is associated with gradual breakdown of the BBB, permitting entry of plasma-derived neurotoxins, inflammatory cells, and microbial pathogens into the CNS. BBB permeability can be visualized directly in AD patients using imaging technologies including dynamic contrast-enhanced and arterial spin labeling magnetic resonance imaging, and recent studies employing these techniques have shown that subtle changes in BBB stability occur prior to deposition of the pathological hallmarks of AD, senile plaques, and neurofibrillary tangles. These studies suggest that BBB disruption may be useful as an early diagnostic marker; however, AD is also accompanied by neuroinflammation, which can complicate these analyses. This review will outline the structural and functional changes to the BBB that occur during AD pathogenesis and highlight current imaging technologies that can detect these subtle changes. Advancing these technologies will improve both the diagnosis and treatment of AD and other neurodegenerative diseases.

## 1. Introduction

The microenvironment of the brain and central nervous system (CNS) is tightly regulated by the blood–brain barrier (BBB), a physiological barrier between the peripheral blood circulation and the brain parenchyma (Engelhardt, 2003). The BBB is a complex, semipermeable neurovascular system that controls the movement of gases, ions, and other molecules essential for brain function, and mediates the clearance of metabolic waste products from the brain (Daneman and Prat, 2015). As it has a significant role in restricting the entry of blood-derived neurotoxins, pathogens, and other damaging components from the peripheral vasculature into the brain, there is ongoing investigation into the interplay between BBB permeability and neurodegeneration (Daneman and Prat, 2015; Sweeney et al., 2018).

Given its importance in proper brain function, studies regarding the loss of BBB integrity and associations with neurodegenerative diseases including Alzheimer’s disease (AD) are becoming more prevalent. Studies suggest that physiological modifications in the cerebral vasculature may contribute to AD pathology and cognitive decline prior to clinically detectable changes in amyloid beta (Aβ) and tau, diagnostic biomarkers of AD (Sweeney et al., 2018, 2019). Identifying disease-specific patterns of BBB permeability may allow it to serve as a potential biomarker for AD and other neurological disorders as well as a targetable therapeutic.

Blood–brain barrier permeability is commonly measured through the ratio of serum to albumin present in cerebrospinal fluid (CSF), with albumin increasing with BBB permeability; however, changes in albumin may not be detectable in prodromal AD and cannot identify specific pathological brain states or localize BBB leakage (Janelidze et al., 2017; Raja et al., 2018; Sweeney et al., 2018, 2019; Erickson and Banks, 2019; Varatharaj et al., 2019; Galea, 2021; Hussain et al., 2021). Furthermore, obtaining CSF samples directly from patients is an invasive procedure and synthesis and cleavage of albumin are affected by age, inflammation, and disease states (Sweeney et al., 2018, 2019; Erickson and Banks, 2019; Varatharaj et al., 2019). To overcome these difficulties, there is ongoing research into the use of *in vivo* imaging techniques, such as dynamic contrast-enhanced magnetic resonance imaging (DCE MRI) and arterial spin labeling MRI (ASL MRI), which may easily detect subtle changes in BBB permeability; however, similar changes are seen as a result of neuroinflammation associated with healthy aging and other neuroinflammatory brain disorders (Verheggen et al., 2020; Hussain et al., 2021). Here, we review the phenotypic changes in the BBB that arise as a result of AD and neuroinflammation. We then highlight select *in vivo* imaging techniques that capture these biological changes, their advantages and challenges, and clinical implications of utilizing these imaging techniques in AD detection and diagnosis.

## 2. Components of the BBB

The structure of the BBB comprises a monolayer of capillary endothelial cells supported by pericytes and astrocytes, together called the neurovascular unit (NVU) (Daneman and Prat, 2015; Sweeney et al., 2018, 2019; Kadry et al., 2020; Verheggen et al., 2020). Ions and molecules move across the BBB through a variety of methods, including passive diffusion and carrier-and receptor-mediated transport, depending on their physical characteristics (Daneman and Prat, 2015; Sweeney et al., 2018; Kadry et al., 2020; Knox et al., 2022).

Blood vessel walls within the CNS are formed by thin, continuous non-fenestrated endothelial cells (EC) that envelop the lumen of the vessel (Daneman and Prat, 2015; Kadry et al., 2020). Because of the compact arrangement of the ECs and low rates of vascular transcytosis, passage of solutes through the BBB is restricted, relying on the expression of specific transporters on ECs (Cabezas et al., 2014; Daneman and Prat, 2015; Knox et al., 2022). The ECs of the BBB are joined by tight junctions (TJ), a dynamic cell junction complex that involves a matrix of transmembrane proteins for structural support and selective permeability, including occludin, claudins, and junction adhesion molecules (Redzic, 2011; Luissint et al., 2012; Bauer et al., 2014; Pulgar, 2019; Lochhead et al., 2020). Together, the interactions of these proteins modulate TJ development, stability, and adhesion.

In addition to ECs, CNS resident pericytes and astrocytes are responsible for maintaining BBB integrity in the steady state. Pericytes attach to the abluminal side of ECs and are responsible for vascular contraction and regulation of cerebral blood flow (Daneman and Prat, 2015; Sweeney et al., 2019; Kadry et al., 2020; Knox et al., 2022). In homeostatic conditions, the association of pericytes and ECs decreases paracellular migration of peripheral immune cells and other inflammatory factors and supports the structure and maintenance of the BBB (Daneman and Prat, 2015; Kadry et al., 2020; Knox et al., 2022). Astrocytes interact with the BBB through the attachment of astrocytic endfeet and are the primary neural component of the NVU, enveloping most of the perivascular surface (Cabezas et al., 2014; Sweeney et al., 2018; Oksanen et al., 2019; Knox et al., 2022). The release of sonic hedgehog (SHH) by astrocytes acts on ECs to modulate TJ protein expression and maintain BBB integrity (Daneman and Prat, 2015; Sweeney et al., 2019; Michinaga et al., 2021). Other factors expressed by astrocytes, including VEGF, glial cell derived neurotrophic factor (GDNF), and angiopoietin-1, mediate angiogenesis and recruitment of TJ proteins, contributing to BBB development and maintenance (Cabezas et al., 2014; Daneman and Prat, 2015; Oksanen et al., 2019). Furthermore, astrocytic endfeet express transporters and channels which regulate pH and the passage of ions to the brain (Cabezas et al., 2014; Liu et al., 2018;Oksanen et al., 2019; Kadry et al., 2020; Knox et al., 2022). For example, ion channels such as the potassium channel Kir4.1 and the water channel aquaporin-4 (AQP-4) balance CNS ionic concentration (Cabezas et al., 2014; Liu et al., 2018; Oksanen et al., 2019; Knox et al., 2022). Astrocytes are also responsible for neurovascular communication and maintenance of cerebral blood flow (Oksanen et al., 2019; Kadry et al., 2020; Knox et al., 2022).

These cells work cooperatively to maintain BBB integrity and prevent infiltration of potentially harmful cells and solutes into the CNS. However, their functional capacity changes in response to AD pathology and inflammatory stimuli, leading to structural alterations and increased permeability.

## 3. Breakdown of the BBB

Elements of the BBB interact to maintain homeostasis and respond dynamically to pathological stressors and disease states. BBB breakdown associated with AD involves physiological changes including the opening of TJs, differential expression of transport proteins, and loss of organization of the NVU, leading to entry of cells and molecules not usually found in the brain (Kadry et al., 2020). These effects have been identified in neuroinflammatory conditions associated with neurodegenerative diseases and CNS infections, as well as with inflammation typical to healthy aging (Knox et al., 2022).

### 3.1. The BBB in healthy aging

Under healthy conditions, the BBB maintains the immune-privileged state of the brain by restricting circulating peripheral immune cells; however, the aging brain exhibits heightened basal levels of proinflammatory cytokines such as interleukin-6 (IL-6), as well as a disequilibrium of anti-inflammatory signals, resulting in activation of CNS-resident microglia (Pachter et al., 2003; Sparkman and Johnson, 2008; Erickson and Banks, 2019). Amplified by the accumulation of age-related dysregulation of other cellular processes, including genome instability and oxidative stress, inflammatory events that activate microglia and recruit leukocytes to the brain promote increased permeability of the BBB (Knox et al., 2022). In particular, IL-1β and tumor necrosis factor (TNF) interact with receptors on ECs of the BBB and modulate BBB permeability in several ways, such as upregulating leukocyte adhesion molecules including P-and E-selectins and ICAM-1 that promote the migration of leukocytes through the BBB (Argaw et al., 2006; Huber et al., 2006; Wang et al., 2014; O’Carroll et al., 2015; Varatharaj and Galea, 2017; Van Dyken and Lacoste, 2018; Galea, 2021; Hussain et al., 2021; Takata et al., 2021). This heightened inflammation also upregulates matrix metalloproteinases (MMPs), increasing the circulation of peripheral immune cells into the brain through degradation of the basement membrane (Varatharaj and Galea, 2017; Van Dyken and Lacoste, 2018; Galea, 2021). MMPs also have a role in reorganization of TJ proteins claudin-5 and occludin, leading to disruption of TJ adhesion, augmented by IL-1β and TNF circulation as well as oxidative stress (Yang et al., 2007; Elahy et al., 2015; Van Dyken and Lacoste, 2018; Galea, 2021; Hussain et al., 2021; Knox et al., 2022). Oligomerization of occludins is further affected by oxidative stress on a molecular level, thus making proper assembly of TJs by occludins sensitive to redox regulation and inflammation (Luissint et al., 2012). Additionally, structural changes such as the loss of pericytes, swelling and detachment of astrocytic endfeet, decreased angiogenesis and vascular density, and glycocalyx degradation further contribute to loss of integrity of the NVU (Varatharaj and Galea, 2017; Brown et al., 2019; Erickson and Banks, 2019; Hussain et al., 2021; Knox et al., 2022). Furthermore, alterations in transport protein expression lead to molecular imbalances within the CNS. For example, downregulation of glucose transport protein GLUT1 and efflux transporters P-glycoprotein and LRP-1 contribute to nutrient starvation and buildup of metabolic waste products (Van Dyken and Lacoste, 2018; Galea, 2021; Hussain et al., 2021). Detachment of astrocytes and decreased expression of AQP-4 on the astrocytic endfeet cause water imbalance within the CNS (Erickson and Banks, 2019; Galea, 2021; Hussain et al., 2021). These changes lead to an overall increased permeability of the BBB and imbalance of the CNS microenvironment, as well as the potential introduction of pathogens and neurotoxins into the brain that can contribute to neurodegenerative processes (Funk et al., 2021; Lotz et al., 2021).

### 3.2. The BBB and AD

Blood–brain barrier vascular disruption often precedes detectable AD symptomatology and neurophysiological changes (Sweeney et al., 2018, 2019). Structural deficits include EC degeneration, detachment of astrocytic endfeet, and pericyte degeneration that cause instability of the vascular architecture, as well as reduced expression of TJ proteins claudin-5 and occludin (Winkler et al., 2011; Zlokovic, 2011; Sweeney et al., 2018, 2019). Data show that deficiency of claudin-5 and occludin in cortical brain regions associated with AD further drives a loss of adhesion between TJs (Yamazaki et al., 2019). Decreased expression of GLUT1 reduces glucose uptake into the brain, leading to hypometabolism, which precedes cognitive impairment (Zlokovic, 2011; Sweeney et al., 2018; Kyrtata et al., 2021). Individuals with AD also exhibit BBB degradation caused by increased activation of the cyclophilin A-MMP-9 pathway (Sweeney et al., 2018, 2019; Hussain et al., 2021). Gradual loss of AQP-4 on astrocytic endfeet as a result of astrogliosis contributes to water imbalance as well as dysregulation of ion homeostasis and solute clearance (Lan et al., 2016; Valenza et al., 2020).

Accumulation of Aβ and tau aggregates accelerates BBB pathologies by promoting aberrant angiogenesis *via* VEGF dysregulation and cerebral amyloid angiopathy (Biron et al., 2011; Zlokovic, 2011; Singh et al., 2017; Steinman et al., 2021). Furthermore, Aβ aggregates promote microglia activation and inflammatory cytokine expression including IL-1β and TNF, which recruit leukocytes to the brain parenchyma, further intensifying BBB breakdown and vessel leakage (Hussain et al., 2021). This is augmented by decreased Aβ clearance from the brain *via* the loss of transporters P-glycoprotein, LRP-1, and AQP-4, as well as increased RAGE-mediated influx of Aβ into the brain (Zlokovic, 2011; Lan et al., 2016; Sweeney et al., 2018, 2019; Oksanen et al., 2019; Valenza et al., 2020; Hussain et al., 2021). Thus, the neuropathological hallmarks of AD and resultant neuroinflammation further diminish BBB integrity, which is already compromised in aged individuals.

Altogether, TJ protein loss, EC, astrocyte, and pericyte degeneration, along with damage to the vasculature from AD pathological aggregates, result in loss of BBB integrity. This allows extravasation of molecules into the brain that lead to microbleeds and other injuries visible on *in vivo* brain scans (Zlokovic, 2011; Daneman and Prat, 2015; Sweeney et al., 2018, 2019).

## 4. Current methods in imaging the BBB

The pathophysiological changes that occur at the BBB in neuroinflammation and AD can be identified *in vivo* by imaging extravasation of specific solutes into the CNS (Sweeney et al., 2018, 2019; Erickson and Banks, 2019; Verheggen et al., 2020; Knox et al., 2022). Here, we describe current imaging techniques that are used to monitor BBB permeability and how they are impacted by neuroinflammation.

### 4.1. Dynamic contrast-enhanced magnetic resonance imaging

A commonly employed imaging technique to measure subtle BBB permeability is DCE MRI, which utilizes continuous scanning prior to and after injection of an intravenously administered gadolinium-based contrast agent (GBCA) to measure the change in gadolinium concentration in the brain (Heye et al., 2014; Montagne et al., 2016a; Varatharaj et al., 2019; Shao et al., 2020). Compared with healthy aging, individuals with AD show increased extravasation of GBCAs into the CNS (Heye et al., 2014; Montagne et al., 2016a). GBCA concentration values are extrapolated from T1-weighted images and analyzed in relation to regional cerebral blood flow and vascular surface area to obtain the transfer coefficient (K_trans_) of permeability per gram of brain tissue (Heye et al., 2014; Montagne et al., 2016a; Varatharaj et al., 2019). K_trans_ can be individually derived for segregated regions of interest to localize BBB permeability (Montagne et al., 2016a; Varatharaj et al., 2019; Shao et al., 2020).

Use of DCE MRI was initially reserved for CNS diseases with robust vascular changes, including ischemic stroke and brain tumors (Heye et al., 2014; Shao et al., 2020). Because of advances in MRI technology that allow for improved temporal and spatial resolution, the extravasation of GBCAs can be observed in individuals with more subtle BBB leakage, including those with diabetes and AD (Heye et al., 2014; Montagne et al., 2015, 2022; Raja et al., 2018; Shao et al., 2020; Verheggen et al., 2020). This imaging technique can identify changes in BBB permeability in cognitively healthy aged individuals (Montagne et al., 2015, 2016a, 2022; Verheggen et al., 2020) and differentiate between BBB breakdown attributed to AD (Montagne et al., 2015; van de Haar et al., 2016a, b), mild cognitive impairment (MCI) (Montagne et al., 2015, 2016a, 2017, 2022), Parkinson’s disease (PD)(Al-Bachari et al., 2020), multiple sclerosis (MS) (Taheri et al., 2013; Cramer et al., 2014; Maranzano et al., 2017), and cerebral small-vessel diseases (cSVD) (Zhang et al., 2017; Uchida et al., 2020; Verheggen et al., 2020).

There are several considerations for the widespread use of DCE MRI as a clinical tool. Although DCE MRI can detect subtle changes in BBB permeability in well-controlled cohorts, differences in scanning equipment, parameters, and data processing between data collection sites can dilute these relatively small changes associated with AD and aging, which are typically one order of magnitude lower than the more robust changes of other disorders like ischemic stroke (Heye et al., 2014; Montagne et al., 2015, 2016a, 2022). It is also widely recognized that GBCAs are relatively toxic, and the large size of the GBCA molecule limits its extravasation when BBB permeability is low (Montagne et al., 2016a,b; Raja et al., 2018; Shao et al., 2019, 2020; Varatharaj et al., 2019). Thus, while DCE MRI is a conventional measure of BBB permeability, researchers seek to better define the sensitivity of the imaging modality and increase options available to patients (Heye et al., 2014; van de Haar et al., 2017; Chagnot et al., 2021; Montagne et al., 2022).

### 4.2. Arterial spin labeling magnetic resonance imaging

Studies have described ASL MRI as an alternative to DCE MRI in identifying subtle BBB leakage (Montagne et al., 2016a; Shao et al., 2020). ASL MRI utilizes arterial spin labeling, magnetically labeling arterial blood water using radiofrequency irradiation to noninvasively exploit water as an endogenous tracer (Detre et al., 2012; Montagne et al., 2016a; Shao et al., 2019, 2020). There are a few approaches to the methodology for ASL, such as continuous and pulsed ASL; however, many studies employ pseudo-continuous ASL, which introduces quick intermittent pulses of radiofrequency over several minutes (Detre et al., 2012; Wolk and Detre, 2012; Shao et al., 2019, 2020; Gold et al., 2021). Readouts of water extraction ratio and cerebral blood flow in relation to capillary permeability-surface area product of water are calculated with arterial transit time and post-labeling delay to obtain the water exchange rate (k_w_) (Shao et al., 2019). Because water molecules are smaller than nearly all contrast agents, its movement across the BBB is sensitive to minute changes in permeability. Furthermore, water is inherently transported across the BBB primarily *via* AQP-4, facilitating its use as a naturally occurring indicator of BBB permeability (Shao et al., 2019, 2020).

In contrast to DCE MRI measurements of vascular permeability, ASL MRI measures cerebral blood flow *via* tissue perfusion (Albrecht et al., 2016; Montagne et al., 2016a). Changes in k_w_ are significantly associated with diminished cognitive performance and decreased Aβ clearance in the hippocampus (Du et al., 2006; Shao et al., 2019; Zheng et al., 2019; Gold et al., 2021). Furthermore, same-subject reproducibility of ASL MRI indicates reliability of the measurement despite low-level BBB leakage (Shao et al., 2019, 2020). Although ASL MRI is a newer imaging modality for studying subtle loss of BBB integrity, it has been validated with other methods, including DCE MRI (Shao et al., 2020; Gold et al., 2021), and studies indicate its clinical potential as a measurement of several neurodegenerative diseases, including AD (Alsop et al., 2000; Dai et al., 2009; Chen et al., 2011; Alexopoulos et al., 2012; Montagne et al., 2016a; van de Haar et al., 2016b; Zheng et al., 2019; Uchida et al., 2022), MCI (Chao et al., 2009; Dai et al., 2009; Alexopoulos et al., 2012; Montagne et al., 2016a), frontotemporal dementia (FTD) (Du et al., 2006; Anazodo et al., 2018), Lewy body disorders (Taylor et al., 2012; Robertson et al., 2016) including PD (Le Heron et al., 2014), and hypercholesterolemia and diabetes, which are known vascular risk factors for AD and cSVD (Shao et al., 2019).

Because the use of ASL MRI in relation to AD neuropathology is still very new, there is a need to validate these metrics with other biomarkers of AD and to characterize disease-specific cerebral blood flow changes longitudinally (Montagne et al., 2016a). For example, the water channel AQP-4 is downregulated early in cerebrovascular degeneration, which may allow detection of early hypoperfusion (Lan et al., 2016; Shao et al., 2020; Valenza et al., 2020). However, loss of TJ adhesion in later pathology may have the opposite effect, which may partly explain disparate results across studies, in which decreased water transfer to the hippocampus, caudate, and thalamus in AD patients was inconsistently observed (Montagne et al., 2016a; Shao et al., 2020; Gold et al., 2021). Further studies are needed to better define regional changes in k_w_ and their correlation to the development of AD.

## 5. Discussion

**Figure 1:**
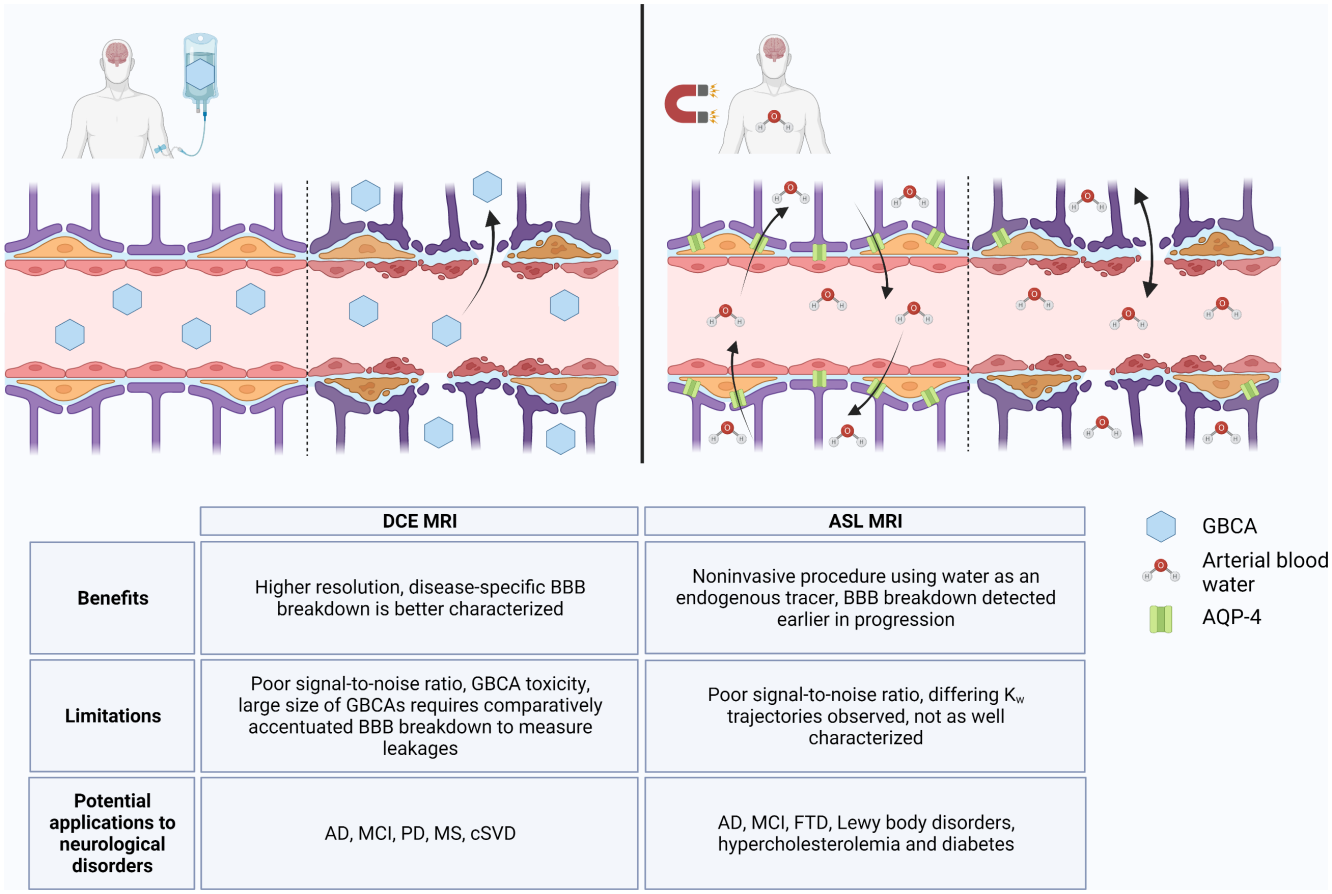
DCE MRI vs. ASL MRI. In an intact blood–brain barrier (BBB), GBCAs are restricted from the brain parenchyma, but with loss of TJs, GBCAs extravasate into the brain *via* paracellular transport, which can be detected by DCE MRI **(left)**. ASL MRI detects changes in water flux through AQP-4 channels, the abundance of which can change in response to normal aging and neurodegenerative processes. With loss of BBB integrity, water flux can also occur *via* paracellular transport **(right)**. Benefits, limitations, and potential applications to diseases for DCE MRI and ASL MRI are listed in table below. Created with BioRender.com.

Alzheimer’s disease is the most prominent type of dementia and the fifth-leading cause of death in individuals over 65 years of age, but there remains a lack of early diagnostic and treatment options for AD (Hussain et al., 2021; Alzheimer’s disease facts and figures, 2022). Measurable changes in Aβ and tau are preceded by BBB breakdown, detection of which may facilitate earlier diagnosis of AD and implementation of disease-modifying therapeutics (Sweeney et al., 2018, 2019). However, BBB breakdown has been shown to also occur throughout normal aging due to neuroinflammatory processes and in other neurological disorders (Verheggen et al., 2020; Hussain et al., 2021). Measurement of CSF albumin ratio can indicate loss of BBB integrity, but its low sensitivity and specificity to disease states limits its use as a predictive biomarker (Janelidze et al., 2017; Sweeney et al., 2018, 2019; Erickson and Banks, 2019; Galea, 2021). Rather, imaging the BBB *via* specialized MRI techniques better characterize regional patterns of BBB breakdown and improve its use in monitoring development of AD. We highlighted two BBB imaging techniques that have been studied in relation to AD pathology: DCE MRI and ASL MRI. As illustrated in Figure 1, both methodologies show potential for diagnostic capability; however, further validation and optimization will be necessary to differentiate between BBB breakdown associated with AD versus other neuroinflammatory disorders.

These technologies are limited by their sensitivity to the subtle BBB changes that occur in early AD pathology and their ability to distinguish among AD, other neurological disorders, and inflammatory processes common in healthy aging. DCE MRI has a comparatively high temporal and spatial resolution, and studies suggest that it can distinguish disease-specific regional BBB breakdown without obfuscation by neuroinflammation (Heye et al., 2014; Montagne et al., 2015, 2022; Raja et al., 2018; Shao et al., 2020). However, the large size of GBCAs and limited extravasation in homeostatic BBB flux limits its use in early disease states during which much smaller BBB changes occur (Shao et al., 2019, 2020; Varatharaj et al., 2019). In contrast, the lower spatial resolution of ASL MRI decreases detection of BBB breakdown in smaller regions such as the hippocampus, but the use of water as a tracer allows ASL MRI to be more sensitive to subtle BBB breakdown (Du et al., 2006; Shao et al., 2020; Uchida et al., 2023). As a smaller molecule than GBCAs, water passes the BBB more easily and can detect loss of AQP-4 in preclinical AD (Shao et al., 2019, 2020). The absence of a GBCA in ASL MRI may also improve availability to GBCA-sensitive patients (Shao et al., 2019). However, ASL MRI will require more in-depth longitudinal studies in order to delineate the differing trajectories of k_w_ due to loss of AQP-4 versus breakdown of TJs (Montagne et al., 2016a; Dickie et al., 2020; Gold et al., 2021).

Changes in cerebral blood flow as measured by ASL MRI are significantly correlated with the presence of Aβ, and DCE MRI studies demonstrate that BBB breakdown precedes accumulation of Aβ and tau and possibly acts independently of AD pathology (Yan et al., 2017; Nation et al., 2019; Montagne et al., 2020; Gold et al., 2021; Uchida et al., 2022). Furthermore, studies have validated the association between *in vivo* imaging measurements from DCE MRI with both ASL MRI and CSF biomarkers such as albumin, indicating the potential of these techniques to predict AD progression (Montagne et al., 2015, 2022; van de Haar et al., 2016b; Varatharaj et al., 2019; Shao et al., 2020; Gold et al., 2021). However, while imaging techniques can be indicative of BBB breakdown related to AD, their use as a solitary diagnostic tool of AD is not definitive and must also be confirmed with additional biomarkers such as Aβ, tau, CSF albumin, and PDGF-B (Montagne et al., 2016a, 2020; Sweeney et al., 2018, 2020; Varatharaj et al., 2019).

Imaging modalities beyond the scope of MRI that may recognize subtle BBB changes include positron emission tomography (PET) scans, which measure decay of radioligands labeling specific target molecules (Omami et al., 2014). For example, [^18^F]-fluoro-2-deoxyglucose (FDG) is a glucose analog transported across the BBB *via* GLUT1. Because GLUT1 expression is downregulated with BBB degeneration, low levels of FDG in the BBB are indicative of BBB pathologies, and can identify AD and other dementias (Shivamurthy et al., 2015; Montagne et al., 2016a; Sweeney et al., 2018, 2019; Bouter et al., 2019; Ou et al., 2019). Additionally, ligands that are substrates of other BBB transport molecules such as P-glycoprotein, an efflux transport protein whose changes in expression are well-characterized with BBB breakdown in AD and neuroinflammation, similarly may demonstrate regional BBB degeneration in patients with AD (van Assema et al., 2012; Deo et al., 2014; Raaphorst et al., 2017; Savolainen et al., 2017; Van Dyken and Lacoste, 2018; García-Varela et al., 2021; Garcia-Varela et al., 2022). These and other radioligands suggest that PET scanning in addition to DCE MRI and ASL MRI may have diagnostic capabilities for neurodegenerative diseases. Other preclinical technologies for visualizing BBB disruption are also being actively investigated, but have not yet been tested in human patients. Some of these technologies include dual-wavelength high-resolution photoacoustic microscopy (Wang et al., 2022), PET-MRI nanoparticles (Debatisse et al., 2020), and quantitative ultra-short time-to-echo contrast-enhanced MRI (Leaston et al., 2021); however, more research is necessary to assess their diagnostic potential.

## 6. Conclusion

Imaging the BBB and associated proteins has the potential to predict AD development. However, subtle BBB breakdown associated with AD, particularly in preclinical AD, can be similar to that in healthy aging due to neuroinflammation or other neurological disorders. As imaging technology and related analysis software continue to improve, image resolution and data extrapolation are better able to detect the subtle BBB breakdown in AD; however, more research is needed to use these techniques to characterize these changes in relation to specific disease states. Discerning the trajectory of BBB breakdown between AD and neuroinflammation necessitates further study of the regional changes that occur, aided by improving imaging methodologies.

## Author contributions

All authors listed have made a substantial, direct, and intellectual contribution to the work and approved it for publication.

## Funding

This work was supported by the National Institutes of Health grant K99/R00 AG053412 to KF and the IDSA Foundation. Its contents are solely the responsibility of the authors and do not necessarily represent the official view of the IDSA Foundation.

## Conflict of interest

The authors declare that the research was conducted in the absence of any commercial or financial relationships that could be construed as a potential conflict of interest.

## Publisher’s note

All claims expressed in this article are solely those of the authors and do not necessarily represent those of their affiliated organizations, or those of the publisher, the editors and the reviewers. Any product that may be evaluated in this article, or claim that may be made by its manufacturer, is not guaranteed or endorsed by the publisher.
